# Chronic Inversion of Uterus—Reduced

**Published:** 1873-11

**Authors:** 


*AMERICAN JOURNAL OBSTETRICS.*

CHRONIC INVERSION OF THE UTERUS OF SIX YEARS STANDING SUCCESSFULLY REDUCED.—Dr. George H. Bixby, of Boston, reports the operation as practiced by Professor James P. White, of Buffalo, New York:

When completely etherized, the patient was placed upon the back across the bed, with the pelvis near the edge, the feet supported by two seated assistants. Prof. White assumed the kneeling posture before the patient. The left hand, well lubricated, was carefully introduced into the vagina, and the fingers slowly insinuated under and about the mass. While the latter was sustained by the palm of the hand, the constricted portion was embraced by the thumb and fingers from opposing sides. With the right hand Dr. White's uterine repository (*Am. Jour. Med. Sci., April,* 1872,) was introduced into the vagina, its concave extremity placed against the fundus or cone of the tumor, and its spring-end rested against the chest of the operator. This done, the right hand, now free, was employed for making counter-pressure above the pubes, or for any other desired purpose. Gentle but unyielding pressure, rendered equable and manageable by the action of the spring, was continued without any variation for thirty minutes, little more force being exerted than that required to compress the spring.

Thirty minutes after the commencement of the operation: Patient in good condition; hemorrhage insignificant; tumor in vagina still firm; constriction unyielding. One hour, from commencement of the operation: Hemorrhage insignificant; tumor more soft; a tendency to collapse, manifested by retarded respiration and pulse, was combated by the injection of an ounce of clear brandy in the rectum, to which the heart's action promptly responded. The repositor was now continued, its action varied by a slow to-and-fro motion of the body upon the spring. One and a half hours: The patient bears the operation well; hemorrhage insignificant; pulse somewhat accelerated; tumor more soft and compressible; a large-size gumelastic rectal bougie substituted for repositor. Two hours and ten minutes: Under the pressure of the bougie and manipulation with the fingers, the constriction suddenly relaxed; and the organ assumed its normal position. The patient rallied from the anæsthesia in less than a quarter-hour; reaction took place slowly. Notwithstanding this prolonged manipulation, neither the vagina nor perineum were in any way injured. The following day there were symptoms of metritis, which rapidly disappeared. One month after operation: She has sat up; good appetite; sleeps well; gains strength; uterus in normal position.

